# Holmium laser enucleation of the prostate: efficacy, safety and preoperative management in patients presenting with anticoagulation therapy

**DOI:** 10.1007/s00345-020-03272-2

**Published:** 2020-06-02

**Authors:** Marina Deuker, Jessica Rührup, Pierre I. Karakiewicz, Maria Welte, Luis A. Kluth, Severine Banek, Frederik C. Roos, Philipp Mandel, Felix K.-H. Chun, Andreas Becker

**Affiliations:** 1 Department of Urology, University Hospital Frankfurt, Theodor-Stern-Kai 7, 60590 Frankfurt am Main, Germany; 2 Cancer Prognostics and Health Outcomes Unit, Division of Urology, University of Montréal Health Center, Montreal, QC Canada

**Keywords:** HoLEP, Anticoagulation, Complications, Bleeding, Transfusion, Clavien–Dindo classification

## Abstract

**Purpose:**

We evaluated efficacy and safety profile of patients with anticoagulation therapy (AT) undergoing holmium laser enucleation of the prostate (HoLEP).

**Methods:**

Within our prospective institutional database (11/2017 to 11/2019), we analyzed functional outcomes and 30-day complication rates of HoLEP patients according to Clavien–Dindo classification (CLD), stratified according to specific AT vs. no AT. Further analyses consisted of uni- and multivariate logistic regression models (LRM) predicting complications.

**Results:**

Of 268 patients undergoing HoLEP, 104 (38.8%) received AT: 25.7% were treated with platelet aggregation inhibitors (PAI), 8.2% with new oral anticoagulants (NOAC) and 4.9% with AT-combinations or coumarins bridged with low molecular weight heparins (LMWH/combination). Patients receiving AT were significantly more comorbid (*p* < 0.01). Pre- and postoperative maximal flow rates, residual void urine and IPSS at 3 months after surgery were invariably improved after HoLEP for patients with/ without AT. Overall complication rate was 19.5% in patients with no AT vs. 26.1% vs. 27.3 vs. 46.2%, respectively, in patients with PAI, NOAC and LMWH/combination (*p* < 0.01). Major complications (CLD ≥ 3b) occurred in 6.1% of no AT patients vs. 4.3% vs. 4.5 vs. 0% in patients with PAI, NOAC and LMWH/combination, respectively (p < 0.01). In multivariate LRM, AT was not significantly associated with higher complication rates, whereas high ASA status (OR 2.2, *p* = 0.04), age (OR 1.04, *p* = 0.02) and bioptical or incidental prostate cancer (OR 2.5, *p* = 0.01) represented independent risk factors.

**Conclusion:**

Despite higher overall complication rates in AT patients, major complications were not more frequent in AT patients. HoLEP is safe and effective in anticoagulated patients.

## Introduction

Bladder outlet obstruction (BOO) represents a frequent condition in older men and its prevalence is increasing with higher age [1, 2]. Especially a rising proportion of patients with cardiovascular diseases require a consequent anticoagulation therapy (AT). Moreover, a rising amount of patients has a regular medication with new oral anticoagulants (NOAC) or with platelet aggregation inhibitors (PAI) [3–5].

For decades transurethral resection of the prostate (TURP) has been considered the gold standard endoscopic approach for subvesical desobstruction [6]. However, it is associated with bleeding complication rates in up to 33% in patients with AT [7]. Safer but equally effective techniques are warranted, such as the holmium laser enucleation of the prostate (HoLEP) [8]. HoLEP is associated with fewer bleeding complications and shorter hospitalization rate compared to TURP and open retropubic enucleation of the prostate [9–11]. Moreover, small cohort studies also suggested the safety and efficacy of HoLEP in patients with AT [12, 13]. We herein report our institutional experience since implementation of HoLEP in 2017 regarding its safety and efficacy in anticoagulated patients. In contrast to older series that mainly included patients taking ASS or Coumarins, our study includes three different groups of AT (PAI, NOAC and low molecular weight heparins (LMWH)/combinations). We hypothesized that HoLEP can be performed safely and effectively in those patients.

## Materials and methods

### Study population

Within our prospective institutional database, 268 consecutive patients who underwent HoLEP for BOO between November 2017 and November 2019 were identified. Patients with urethral strictures were excluded. Patients were stratified into four groups depending on the kind of perioperative AT vs. no AT. Acetylsalicylic acid (ASS) and platelet aggregation inhibitors (PAI, such as clopidogrel) were grouped as PAI, the second group consisted of new oral anticoagulants (NOAC; such as dabigatran, a direct thrombin inhibitor, or oral direct factor Xa inhibitors, such as rivaroxaban or apixaban), patients with low molecular weight heparins (LMWH), or bridged with LMWH as substitution for Vitamin K antagonists (Coumarins) or any combination agents were registered among “LMWH/combination”.

### Perioperative management of patients with AT

Patients, who took Coumarins, stopped taking the medication 10 days before HoLEP and were bridged with LMWH during the perioperative period. Patients taking NOAC, stopped taking medication 48 h before surgery without replacement; postoperatively they were treated with LMWH in semi-therapeutic scheme for 14 days. Patients on ASS did not pause ASS and patients with other PAI were either changed to ASS or temporarily paused the medication. All patients had an INR < 1.5 and at least 80,000 thrombocytes/µl at time of surgery. However, in agreement with the treating general physician or cardiologist, also other schemes were individually fitted in accordance with specific patient requirements.

All surgical procedures were performed with the Olympus, OES Pro Laser Resectoscope, a high power (120 W) holmium laser generator (MOSES Pulse 120H, Lumenis), a 550 nm laser fiber (Lumenis, Slim Line) and a morcellator (Versacut, Lumenis). Operations were performed in one-, two- or three-lobe technique. HoLEP was performed by three experienced senior HoLEP-surgeons. An overview of the individual surgical steps can be viewed online at GeSRU StepS (https://www.gesru.de/fuer-assistenzaerzte/fortbildung/gesru-steps/steps-video/) as a surgical tutorial video.

Postoperative complications were recorded according to the Clavien–Dindo (CLD) classification system using a graduation from CLD I to CLD V [14]. Complications occurring within 30 days after the procedure were recorded as early complications. Major complications were defined as CLD equal or greater IIIb. The follow-up was scheduled by the use of systematic questionnaires that were sent to the patients prospectively after 4 weeks, 3 months and one year. Catheter status and—if applicable—uroflow and post-void residual urine were examined in all patients before discharge. Follow-up, including IPSS was available for 26% (*n* = 70).

### Statistical analysis

Descriptive statistics included frequencies and proportions for categorical variables. Means, medians, and interquartile-ranges (IR) were reported for continuously coded variables. The Chi-square test was used to assess the statistical significance in proportions’ differences. The *t* test and Kruskal–Wallis test examined the statistical significance of means’ and medians’ differences. Multivariate logistic regression models (LRM) tested the effect of AT on complications.

In all statistical analyses, R software environment for statistical computing and graphics (version 3.6.1) was used. All tests were two-sided with a level of significance set at *p* < 0.05 (bold font in tables). Ethical approval was obtained from the local ethics committee at the University Hospital of Frankfurt. All included patients gave informed written consent.

## Results

### Descriptive characteristics of the study population

In our institutional database, we identified 268 eligible patients, who underwent HoLEP at the University Hospital Frankfurt from November 2017 to November 2019. Of those, 104 patients (38.8%) were treated with AT. 69 patients (25.7% of the total cohort) were treated with PAI (among those were 66 patients with ASS-monotherapy) vs. 22 patients (8.2%) received NOAC vs. 13 patients (4.9%) with LMWH/combination (Table 1). Patients with AT were significantly older and showed more comorbidities (higher ASA status, *p* < 0.01). Prostate volume measured by TRUS (median: 79 vs. 65 vs. 80 vs. 60 ml) and PSA (median: 5.3 vs. 4.2 vs. 4.8 vs. 3.9 ng/ml) did not differ significantly between the subgroups.

**Table 1:** Preoperative and perioperative characteristics of 268 HoLEP patients stratified according to comedication with anticoagulative therapy (AT)

Variable	Cat/Stat	Overall, *n* = 268 (%)	No AT, *n* = 164 (61.2)	PAI, *n* = 69 (25.7)	NOAC, *n* = 22 (8.2)	LMWH/comb., *n* = 13 (4.9)	*p* value
Preoperative							
Age (years)	Median (IQR)	70 (63–76)	67.5 (61–74)	73 (67–78)	73.5 (65–78)	74 (71–79)	**<0.01**
	Mean (STE)	69.5 (0.6)	67.5 (0.674)	72.4 (0.916)	72.6 (1.541)	73.9 (1.603)	**<0.01**
PSA (ng/ml)	Median (IQR)	4.9 (2.8–8.6)	5.3 (2.8–9.4)	4.2 (2.4–8.2)	4.8 (2.1–6.3)	3.9 (3.6–4.9)	0.2
	Mean (SD)	8.5 (1.1)	9.6 (1.241)	7.9 (1.846)	5 (0.728)	4.3 (0.801)	**<0.01**
TRUS (ml)	Median (IQR)	73 (54–100)	79 (55–104)	65 (50–98.5)	80 (57–88.8)	60 (50–95)	0.06
	Mean (SD)	82.7 (3.186)	87.3 (3.353)	76.1 (4.862)	75.6 (5.04)	72.6 (9.239)	0.06
ASA	I	17 (6.3)	15 (9.1)	2 (2.9)	0 (0)	0 (0)	**<0.01**
	II	147 (54.9)	122 (74.4)	21 (30.4)	4 (18.2)	0 (0)	
	III	101 (37.7)	26 (15.9)	45 (65.2)	17 (77.3)	13 (100)	
	IV	3 (1.1)	1 (0.6)	1 (1.4)	1 (4.5)	0 (0)	
Perioperative							
Catheter time (days)	Median (IQR)	2 (2–2)	2 (2–3)	2 (2–3)	2 (2–2)	2 (2–2)	0.07
	Mean (SD)	2.2 (0.04)	2.2 (0.044)	2.4 (0.118)	2.2 (0.146)	2 (0)	0.2
Enucleation volume (g)	Median (IQR)	51.3 (29–84)	56.5 (32.2–86.8)	45.5 (23.2–64.8)	46 (29.4–65.8)	39 (33.7–53.9)	**0.04**
	Mean (SD)	61.04 (3.506)	66.4 (3.797)	52.2 (4.522)	56 (8.789)	48.8 (9.71)	**0.02**
Operative time (min)	Median (IQR)	87.5 (60–120)	90 (61.8–120)	85 (62–120)	74 (60–138.2)	85 (53–105)	0.2
	Mean (SD)	98.37 (3.08)	100.4 (3.907)	93.2 (4.671)	108.3 (15.726)	83.4 (11.058)	0.2
Velocity of tissue retrieval (g/min)	Median (IQR)	0.6 (0.3–0.9)	0.7 (0.4–0.9)	0.5 (0.3–0.8)	0.6 (0.3–0.9)	0.6 (0.4–1)	0.08
	Mean (SD)	0.7 (0.03)	0.7 (0.032)	0.6 (0.045)	0.6 (0.088)	0.7 (0.124)	0.05
Prostate carcinoma	No	221 (82.5)	139 (84.8)	55 (79.7)	16 (72.7)	11 (84.6)	0.9
	Incidental	29 (10.8)	15 (9.1)	9 (13.0)	5 (22.7)	0 (0)	
	Bioptical	14 (5.2)	7 (4.3)	4 (5.8)	1 (4.5)	2 (15.4)	

*PAI* platelet aggregation inhibitors, *NOAC* new oral anticoagulants, *LMWH/comb.* low molecular weight heparins or combination of AT, *IQR* interquartile range, *SD* standard deviation, *TRUS* transurethral prostate volume, *ASA* American Society of Anesthesiologists’ score

## Perioperative outcomes

Perioperative outcomes did not show major differences according to AT (Table 1). Catheter time (median 2 days in all subgroups, *p* = 0.07), operative time (85–90 min, *p* = 0.2) and velocity of tissue retrieval (operative time divided by enucleation volume: 0.5–0.7 g/min, *p* = 0.08), as well as the incidence of prostate carcinoma did not show statistically significant differences. Conversely, the mean enucleation volume was significantly higher in no AT patients (*p* = 0.04).

### Postoperative outcomes

**Fig. 1:**
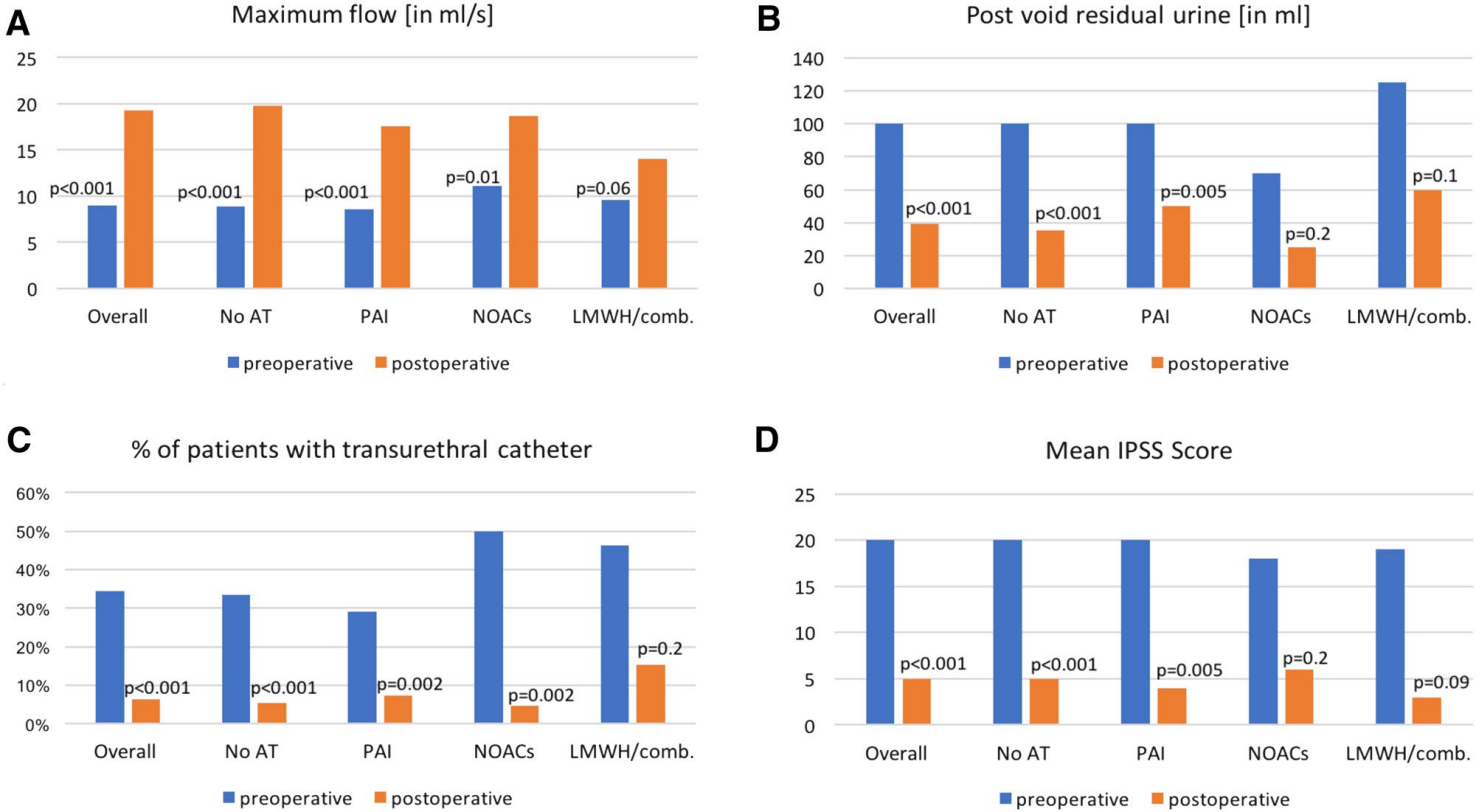
Preoperative and postoperative rates of **a** maximum urine flow^#^, **b** post void residual urine^#^, **c** number of patients with catheter at discharge and **d** IPSS (International prostate symptoms score) at 3 months^#^, stratified according to comedication with anticoagulation therapy (AT). ^#^median is reported. *PAI *platelet aggregation inhibitors, *NOACs* new oral anticoagulants, *LMWH/comb.* low molecular weight heparins or combination of AT

Preoperative and postoperative rates of maximal flow in uroflowmetry, residual void urine, number of patients with transurethral catheter at discharge and IPSS (International prostate symptoms score) at 3 months after surgery were invariably improved after HoLEP in each AT group (Fig. 1a–d). Due to sample size limitations, a statistically significant effect could not consistently be confirmed in NOAC and LMWH/combination patients in each subanalysis.

### Complication rates according to Clavien–Dindo classification

Complication rates were assessed according to CLD classification system [15] (Table 1). Significant differences were recorded for overall early (30 days) complication rates with 19.5% in no AT patients vs. 26.1% vs. 27.3 vs. 46.2%, respectively, in PAI, NOAC and LMWH/combination patients (*p* < 0.01).

In stratified analyses according to minor vs. major complications, minor complications were significantly more frequent in patients with AT compared to patients with no AT. In contrast, the rate of major complications was not higher in patients receiving AT (6.1% of no AT patients vs. 4.3% vs. 4.5 vs. 0% of, respectively, PAI, NOAC and LMWH/combination patients, *p* < 0.01).

### Logistic regression model predicting complication rates

An exploratory univariate logistic regression model analyzed early complication rates according to AT, ASA status, age, operative time, bioptical or incidental finding of prostate carcinoma (PCa) and prostate enucleation volume. Perioperative treatment with LMWH/combination (odds ratio [OR] 3.5, *p* = 0.03), high ASA status (OR 2.6, *p* < 0.01), age (OR 1.1, *p* < 0.01) and bioptical/incidental PCa (OR 2.9, *p* < 0.01) were identified as univariate predictors of complications (Table 2). Conversely, in multivariate analyses adjusted for the given risk factors, AT was no longer significantly associated with higher complication rates, whereas high ASA status (OR 2.2, *p* = 0.04), age (OR 1.04, *p* = 0.02) and bioptical/incidental PCa (OR 2.5, *p* = 0.01) were confirmed as independent predictors of overall complications (Table 3).

**Table 2:** Early complication rates according to Clavien–Dindo classification (CLD) of 268 HoLEP-patients, stratified according to comedication with anticoagulative therapy (AT)

Variable	Cat/Stat	Overall, *n* = 268 (%)	No AT, *n* = 164 (61.2)	PAI, *n* = 69 (25.7)	NOAC, *n* = 22 (8.2)	LMWH/comb., *n* = 13 (4.9)	*p* value
30 days-complication rates	No complication	206 (76.9)	132 (80.5)	51 (73.9)	16 (72.7)	7 (53.8)	**<0.01**
	CLD-I	25 (9.3)	13 (7.9)	7 (10.1)	3 (13.6)	2 (15.4)	
	CLD-II^a^	15 (5.6)	5 (3)	6 (8.7)	2 (9.1)	2 (15.4)	
	Transfusion	1 (0.4)	0 (0)	0 (0)	0 (0)	1 (7.7)	
	CLD-IIIa	7 (2.6)	4 (2.4)	2 (2.9)	0 (0)	1 (7.7)	
	CLD-IIIb	13 (4.9)	9 (5.5)	3 (4.3)	1 (4.5)	0 (0)	
	CLD-IV/V	1 (0.4)	1 (0.6)	0 (0)	0 (0)	0 (0)	
Minor vs. major (CLD ≥ IIIb) Complication	No	206 (76.9)	132 (80.5)	51 (73.9)	16 (72.7)	7 (53.8)	**<0.01**
	Minor	48 (17.9)	22 (13.4)	15 (21.7)	5 (22.7)	6 (46.2)	
	Major	14 (5.2)	10 (6.1)	3 (4.3)	1 (4.5)	0 (0)	
Specific complications							
CLD-I	Urinary retention	13 (4.9)	7 (4.2)	5 (7.2)	2 (9.1)	1 (7.7)	**–**
	Macrohematuria	10 (3.7)	5 (3.0)	2 (2.9)	0 (0)	1 (7.7)	**–**
	Other	2 (0.7)	1 (0.6)	0 (0)	1 (4.5)	0 (0)	**–**
CLD-II	Antibiotics	13 (4.9)	4 (2.4)	5 (7.2)	2 (9.1)	2 (15.4)	**–**
	Transfusion	1 (0.4)	0 (0)	0 (0)	0 (0)	1 (7.7)	**–**
	Other medication	2 (0.7)	1 (0.6)	1 (1.4)	0 (0)	0 (0)	**–**
CLD-IIIa	SPC	7 (2.6)	4 (2.4)	2 (2.9)	0 (0)	1 (7.7)	**–**
CLD-IIIb	Revision in 12 h (bleeding)	5 (1.9)	4 (2.4)	0 (0)	1 (4.5)	0 (0)	**–**
	<30 days revision for bleeding	4 (1.5)	2 (1.2)	1 (1.4)	0 (0)	0 (0)	**–**
	<30 days revision for other	4 (1.5)	3 (1.8)	2 (2.9)	0 (0)	0 (0)	**–**
CLD-IV/V	Death	1 (0.4)	0 (0)	0 (0)	0 (0)	0 (0)	**–**

*PAI* platelet aggregation inhibitors, *NOAC* new oral anticoagulants, *LMWH/comb*. low molecular weight heparins or combination of AT, *SPC* suprapubic catheter

^a^Other than transfusion

**Table 3.** (A) Exploratory analysis with univariable logistic regression model predicting early (30 days) complication rates according to anticoagulation therapy (AT), ASA status, age, operative time, history of prostate carcinoma and prostate enucleation volume; (B) multivariable logistic regression model predicting early (30 days) complication rates according to univariable significant covariables: anticoagulation therapy, ASA status, age and history of prostate carcinoma

Variable	Odds ratio			Confidence interval (2.5–97.5%)	*p* value
(A) Univariable predictors of complications					
No AT PAI NOACs LMWH/comb		Ref 1.5 1.5 3.5	– 0.7–2.8 0.5–4.1 1.1–11.4		– 0.3 0.4 **0.03**
ASA I/II ASA III/IV		Ref 2.6	– 1.4–4.6		– **<0.01**
Age		1.1	1.0–1.1		**<0.01**
Operative time		1.0	1.0–1.0		0.3
No prostate carcinoma (PCa) Bioptical or incidental PCa		Ref 2.9	– 1.4–5.8		– **<0.01**
Prostate enucleation volume		1.0	1.0–1.0		0.7
(B) Multivariable predictors of complications					
No AT PAI NOACs LMWH/comb		Ref 0.8 0.7 1.5	– 0.3–1.7 0.2–2.0 0.4–5.6		– 0.5 0.5 0.6
ASA I/II ASA III/IV		Ref 2.2	– 1.0–4.6		–
					**0.04**
Age		1.04	1.0–1.1		**0.02**
No PCa Bioptical or incidental PCa		2.5	1.2–5.1		**0.01**

*PAI* platelet aggregation inhibitors, *NOAC* new oral anticoagulants, *LMWH/comb.* low molecular weight heparins or combination of AT

## Discussion

We reported our institutional experience regarding safety and efficacy of HoLEP in anticoagulated patients. Our analyses yielded several important observations.

First, AT therapy is widely used in candidates for surgical desobstruction for BOO. More than one third (38.8%) of BPH patients undergoing HoLEP were treated with AT therapy perioperatively. 25.7% of AT patients received PAI, 8.2% received NOAC (8.2%) and 4.9% LMWH/combination. AT patients were older and showed a higher burden of multimorbid conditions (high ASA status). With an aging population and widely available medical care on a high level in western countries more and more patients with polypharmacotherapy are seeking operative treatments [16, 17]. Thus, safe surgical modalities, such as HoLEP are warranted.

Second, we confirmed a favorable safety profile of HoLEP in patients regardless of AT status. Even though we observed a higher incidence of overall complications for patients with perioperative AT, this difference is exclusively attributed to a higher incidence of minor complications according to the CLD classification. Accordingly, no worse outcomes were observed regarding the rate of blood transfusions or major complications such as operative revisions (0–4.3% in AT vs. 6.1% in no AT patients). A large study reporting the outcome of 2,178 patients (of whom 245 either took NOACs or Coumarins), analyzed by Becker et al. [18] in 2019, also reported a higher overall complication rate in patients taking AT. However, they did not classify according to Clavien–Dindo classification. Moreover, Zheng et al. [19] conducted a systematic meta analysis of overall nine studies analyzing the impact of AT on perioperative outcomes after HoLEP. They also found a significantly higher complication rate (including blood transfusion, bladder tamponade, urinary retention) in patients taking AT.

Various large series on HoLEP have reported its superiority over TURP with special regard to a lower risk of bleeding complications [20–22]. A potential explanation for the superior risk profile of HoLEP is, that prostatic vessels are dissected and coagulated only once during the enucleation process. However, considerably less detail is available regarding HoLEP and the perioperative use of AT [13, 23, 24] with most of the studies addressing only transfusion rates. In our study cohort, only one patient (0.4% of the study cohort/ 1% of AT patients) required a blood transfusion. This patient belonged to the LMWH/combination group (Coumarin, bridged with LMWH). In comparison to our very low transfusion rate, transfusion rates in previous studies had a vast range between 0% and 14.7% for patients taking AT: the largest reported series of HoLEP in AT patients (*n* = 116 patients with AT) by El Tayeb et al. [23] reported generally higher transfusion rates compared to the current series. However, their transfusion rates did not differ according to AT vs. no AT status (3.5 vs.1.6%, *p* = 0.13). One smaller study (*n* = 52) by Bishop et al. [13] showed a significant higher transfusion rate in AT patients (7.7 vs 0%, *p* = 0.03). Another two institutional studies reported no transfusion (*n* = 39) [24] and up to 14.7% (*n* = 83) according to specific AT agents [12].

Neuville et al. stated a significantly longer length of stay and longer length of bladder irrigation in patients taking AT, while patients taking PAI did not show significant differences to the no AT cohort. Only three studies reporting reoperation rates are currently available: Tyson et al. [24] reported on a staged procedure due to obscured vision by hematuria in 2/39 (5.1%) patients with AT as opposed to 5/37 (13.5%) patients without AT. Elzayat et al. [12] reported reoperation rates in 3.6% (*n* = 3/83) of AT patients due to bleeding refractory to transfusion and El Tayeb et al. [23] reported reoperation rates in 1.9% (2/116) of AT patients for clot removal. Those results are in line with our observed reoperation rates in AT patients (0–4.5%). It is of note that our study is the second largest out of three studies to report differentiated complication rates according to the Clavien–Dindo classification of complications.

Furthermore, we performed logistic regression models predicting complication rates and found AT to be univariately associated with higher complication rates (OR 3.5, *p* = 0.03 for LMWH/combination). Nevertheless, also high ASA status (OR 2.6, *p* < 0.01), age (OR 1.1, *p* < 0.01), and prostate carcinoma (OR 2.9, *p* < 0.01) were associated with univariably higher complication rates. Moreover, after adjusting for high ASA status and higher age, that were significantly more prevalent within AT patients, the predictive effect of complications disappeared for AT. Thus, it can be concluded that HoLEP can safely be performed in AT patients without higher rates of meaningful complications and overall very low transfusion rates regardless of AT status.

Third, regarding peri- and postoperative outcomes, no major differences between no AT and AT patients were recorded regarding operative time and catheter time in our analyses. This is conflicting with two other reports [13, 23] stating longer length of stay for AT patients and El Tayeb et al. [23] even stating a shorter operation time in patients taking AT. It is of note that in these reports, median length of stay was 1 day as opposed to 2 days, representing our institutional standard before catheter removal and discharge. Boeri et al. also affirmed a comparatively longer hospital stay and catheter maintenance, while they did not see any difference regarding operation time [25].

Interestingly, we observed a lower enucleation volume in the AT group, whereas operation time was comparable between the groups. This “volume effect” might be related to preferential use of HoLEP in patients with AT compared to TUR-P (patient selection). However, the lower enucleation volume did not translate into significant differences in operative time or differences in efficacy of enucleation, as displayed by the velocity of tissue retrieval. Taken together, our institutional standard seems to offer a safe approach for patients with and without AT.

Forth, immediate postoperative assessment of maximum flow and residual urine after removal of the transurethral catheter invariably improved after HoLEP in each AT group. Due to sample size limitations, a significant effect could not consistently be confirmed in NOAC and LMWH/combination patients in each subanalysis. However, even in these small groups, the medians before and after HoLEP improved clinically meaningfully (maximum flow pre- vs. postoperatively 11.1. vs. 18.7 ml/s in NOACs and 9.6 vs. 14.0 ml/s in LMWH/combination and residual void urine 70 vs. 25 ml in NOACs and 125 vs. 60 ml in LMWH/combination). Moreover, proportion of successful postoperative voiding trial improved in all AT groups except for LMWH/combination patients. In this group proportion of successful postoperative voiding trial nevertheless clinically meaningfully improved (46.2 to 15.4%) despite not being statistically significant due to small sample size (6, respectively, 2 of 13). Similarly, IPSS at 3 months after surgery improved clinically meaningfully (pre- vs. postoperatively 18 vs. 6 in NOACs and 19 vs. 3 in LMWH/combination) despite not being statistically significant due to small sample size. It can be postulated that HoLEP in AT patients is equally effective as in no AT patients. Moreover, it is of note that several studies [26, 27] reported on ongoing improvements within the first 6 months after HoLEP and thus, these very early results at discharge will invariably be subject to further improvements.

Taken together, patients with AT represent a growing proportion of contemporary patients. The complication rate of HoLEP performed under AT did not differ according to AT status after multivariate adjustment for covariates as age and ASA status. It is of note that this is the first study to report complication rates according to CLD classification and four different groups of AT.

However, several limitations apply: Despite providing one of the largest series of AT patients in the context of HoLEP, the still limited number of cases (*n* = 104 AT patients) restricted depth of subgroup analyses according to specific AT agents. Thus, future research on this topic should focus on prospective multicenter studies further elucidating the safety and efficacy of HoLEP in AT patients. Secondary, rates of complications were relatively low (overall 76.9% of patients without any complication) impeding further sub analyses, e.g., risk prediction of major complication. Moreover, we did not capture any laboratory results in our database, nor the full medication list of each patient. In consequence we cannot account for the amount of Hb drop or other changes in laboratory findings, nor could we assess potential drug interactions, that might have predisposed some patients at a higher bleeding risk. However, we are confident, that transfusion rate represents indeed a very robust and clinical meaningful endpoint for bleeding complications. In addition, we did not perform standardized assessment of the patients’ comorbidities with the Charlson Comorbidity Index; however, as a proxy for comorbidities, we reported the ASA status of our patients. Moreover, not all patients responded to the standardized follow-up questionnaires, in consequence, especially the IPSS-values are not devoid of missing data (*n* = 70). Furthermore, our prospective database may be influenced by a negative selection bias regarding the admittance of patients with a particularly high perioperative risk to our tertiary care university centre. Nevertheless, we included all HoLEP patients since its implementation at our institution to allow most comprehensive analyses.

## Conclusion

Despite higher minor complication rates in AT patients, major complications were not more frequent in AT patients. After adjustment for covariates as age and ASA status, complication rate did not differ according to AT status. HoLEP is a safe and effective technique for enucleation of the prostate in anticoagulated patients.

## Availability of data and material

The data is available for requests.
